# New Clinical Implications of Opiate Maintenance Treatments

**DOI:** 10.5812/ijhrba.6532

**Published:** 2012-07-25

**Authors:** Gonzalo Haro

**Affiliations:** 1 Program of Dual Pathology. Hospital Provincial University, Castellon, Spain

**Keywords:** Methadone, Buprenorphine, Testosterone

*Dear Editor*

Opiate addiction is a chronic mental illness with severe repercussion in personal and public health. Various medicaments have been subjected to research in order to find a proper treatment, methadone is the most and wide used drug, buprenorphine/naloxone is the most recent ones, but other choices have been proposed such as prescribing heroin (1). The mentioned treatments, are considered as maintenance ones, because, the chronic illnesses need a longtime approach. Other possibilities have been studied, for example the detoxification (conventional or ultra-rapid) as well as the use of opiate antagonist as naltrexone. On the other hand, a long period of dis habituation or relapse prevention is required after detoxification of all sorts of addictions, including opiate dependency.

On this second phase, psychological therapies are very important, as well as social and family support. Most patients need during dis habituation (of heroin addiction) a preliminary period of opiate maintenance with methadone or buprenorphine/naloxone (2).

Side effects, as well as notwell-known effects, of drug and treatments are a very interesting goal of research. The hypogonadotrophic effect of opioids can be observed even after the administration of a single opioid dose, both in laboratory animals and in humans (3). Other studies have reported that methadone and buprenorphine are opioids with effects on spermatogenesis (4) and secondary sex organs. It seems probable that these drugs could affect testes volume.

Otherwise, those effects have quantitative differences depending on the opiate as showed in the paper titled “A quantitative and qualitative study of rat testis following administration of methadone and buprenorphine” (5).

Heidari et al. observed Buprenorphine treatment is more appropriate for treating opiate addiction in male rats since it does not affect normal testicular structure and function (5). Those results should be considered by all the professionals involved in addictions and their co-morbidities (dual pathology) research projects and treatment, not only because of the consequences of this side effect on sexuality or reproduction, but also how the testosterone deficiency is related to the not well-known effects of opioid maintenance treatments, as its antipsychotic properties or its influence on personality dimensions as reward dependence on opiate dependents type II or antisocial (6).
